# The AKT modulator A-443654 reduces α-synuclein expression and normalizes ER stress and autophagy

**DOI:** 10.1016/j.jbc.2021.101191

**Published:** 2021-09-11

**Authors:** Mandi Gandelman, Warunee Dansithong, Stephen C. Kales, Sharan Paul, Gentrie Maag, Erika Aoyama, Alexey Zakharov, Ganesha Rai, Thomas Dexheimer, Brooke M. Whitehill, Hongmao Sun, Ajit Jadhav, Anton Simeonov, Mark J. Henderson, Duong P. Huynh, Stefan M. Pulst, Daniel R. Scoles

**Affiliations:** 1 Department of Neurology, University of Utah, Salt Lake City, Utah, USA; 2 Department of Neurology, National Center for Advancing Translational Sciences (NCATS), Rockville, Maryland, USA; 3 Department of Pharmacology and Toxicology, Michigan State University, East Lansing, Michigan, USA

**Keywords:** Parkinson disease, alpha-synuclein (α-synuclein), SNCA, AKT, A-443654, high-throughput screening (HTS), endoplasmic reticulum stress (ER stress), STAU1, staufen1, autophagy, CRISPRi, CRISPR interference, dCas9, deactivated Cas9, DCLK1, doublecortin like kinase 1, DMEM, Dulbecco's modified Eagle medium, ER, endoplasmic reticulum, GF-AFC, Gly-Phe-7-amino-4-trifluoromethylcoumarin, iPSC, induced pluripotent stem cell, mTOR, molecular target of rapamycin, PD, Parkinson’s disease, UPR, unfolded protein response

## Abstract

Accumulation of α-synuclein is a main underlying pathological feature of Parkinson’s disease and α-synucleinopathies, for which lowering expression of the α-synuclein gene (*SNCA*) is a potential therapeutic avenue. Using a cell-based luciferase reporter of *SNCA* expression we performed a quantitative high-throughput screen of 155,885 compounds and identified A-443654, an inhibitor of the multiple functional kinase AKT, as a potent inhibitor of *SNCA*. HEK-293 cells with CAG repeat expanded *ATXN2* (ATXN2-Q58 cells) have increased levels of α-synuclein. We found that A-443654 normalized levels of both *SNCA* mRNA and α-synuclein monomers and oligomers in ATXN2-Q58 cells. A-443654 also normalized levels of α-synuclein in fibroblasts and iPSC-derived dopaminergic neurons from a patient carrying a triplication of the *SNCA* gene. Analysis of autophagy and endoplasmic reticulum stress markers showed that A-443654 successfully prevented α-synuclein toxicity and restored cell function in ATXN2-Q58 cells, normalizing the levels of mTOR, LC3-II, p62, STAU1, BiP, and CHOP. A-443654 also decreased the expression of DCLK1, an inhibitor of α-synuclein lysosomal degradation. Our study identifies A-443654 and AKT inhibition as a potential strategy for reducing *SNCA* expression and treating Parkinson’s disease pathology.

Parkinson’s disease (PD) is the second most common neurological disorder, and currently there are no treatments that modify disease progression. Mutations in the *SNCA* gene can cause autosomal dominant Parkinson’s disease 1 (PARK1), and duplications or triplications of wildtype *SNCA* are also associated with sporadic PD cases (1, 2, 3, 4, 5). Aggregated and misfolded α-synuclein is the main component of Lewy bodies occurring in the substantia nigra pars compacta, which are a hallmark feature of PD, and is associated with dementia with Lewy bodies, diffuse Lewy body disease, and glial cytoplasmic inclusions in multiple system atrophy collectively known as the synucleinopathies (6). Evidence of *SNCA* multiplications and constitutive phosphorylation in synucleinopathies suggests overexpression and toxic gain of function as a causative factor of disease, supported by increased neuronal cell death being associated with altered *SNCA* expression in patients with PD and in model systems (7, 8, 9, 10, 11, 12, 13).

Oligomeric forms of α-synuclein are widely thought to drive toxicity as they modify many normal cellular functions, including mitochondrial function, endoplasmic reticulum (ER) stress, synaptic function, proteasome function, and autophagy, which can spread through the nervous system (14). Moreover, dissociation of fibrillar α-synuclein may lead to elevation of toxic α-synuclein oligomers, leading to uncertainty about which α-synuclein species is best to target to reduce toxicity (15, 16). Ultimately, evidence indicates that an approach for lowering the overall expression of α-synuclein would be promising as a therapeutic for PD and other α-synucleinopathies.

Studies using animal models support that therapeutics lowering overall *SNCA* expression may improve PD phenotypes, even when treatment begins after disease onset: In a mouse model of Lewy body disease, with inducible A53T-*SNCA* expression, transgene suppression improved learning and memory phenotypes and reduced Lewy body-like aggregates in cortex and hippocampus (17). Similarly, in A30P-*SNCA* inducible mice, abnormal olfactory bulb dopamine levels and behavioral deficits were restored by transgene suppression (18).

In order to identify small molecule *SNCA* inhibitors we performed a quantitative high-throughput screen to identify compounds lowering *SNCA* expression. The screening assay was generated using genome editing to insert the luciferase gene downstream and in frame of the *SNCA* gene in HEK-293 cells (19). We screened 155,885 compounds and identified multiple hits, including a known AKT inhibitor A-443654. A-443654 lowered *SNCA* expression in PD patient–derived fibroblasts and in dopaminergic neurons differentiated from PD patient–derived induced pluripotent stem cells (iPSCs). In addition, A-443654 normalized α-synuclein monomers and oligomers, *SCNA* expression, and autophagy and ER stress markers in HEK-293 cells. Our study identified A-443654 as a small molecule regulator of α-synuclein levels and toxicity in multiple cellular models of PD.

## Results

### Luciferase assay validation

Previously we engineered a cell line that expresses luciferase from the *SNCA* locus, HEK-293-SNCA-luc, which demonstrated robust response to *SNCA* siRNA or a small molecule luciferase inhibitor (19). To further validate that luciferase activity specifically reflects transcriptional activity from the *SNCA* locus, we performed a clustered regularly interspaced short palindromic repeat (CRISPR) interference (CRISPRi) assay. A single guide RNA was used to direct a nuclease deactivated Cas9 (dCas9) tethered to the KRAB transcriptional repressor to just downstream of the *SNCA* transcriptional start site (TSS) (20). Cotransfection of CRISPRi plasmids in HEK-293-SNCA-luc cells resulted in 45% reduction of luciferase expression from the *SNCA* locus compared with untransfected cells, whereas transfection of CRISPRi plasmids in HEK-293-CMV-luc control cells did not inhibit luciferase expression (Fig. S1).

### Quantitative HTS and identification of A-443654

**Figure 1:**
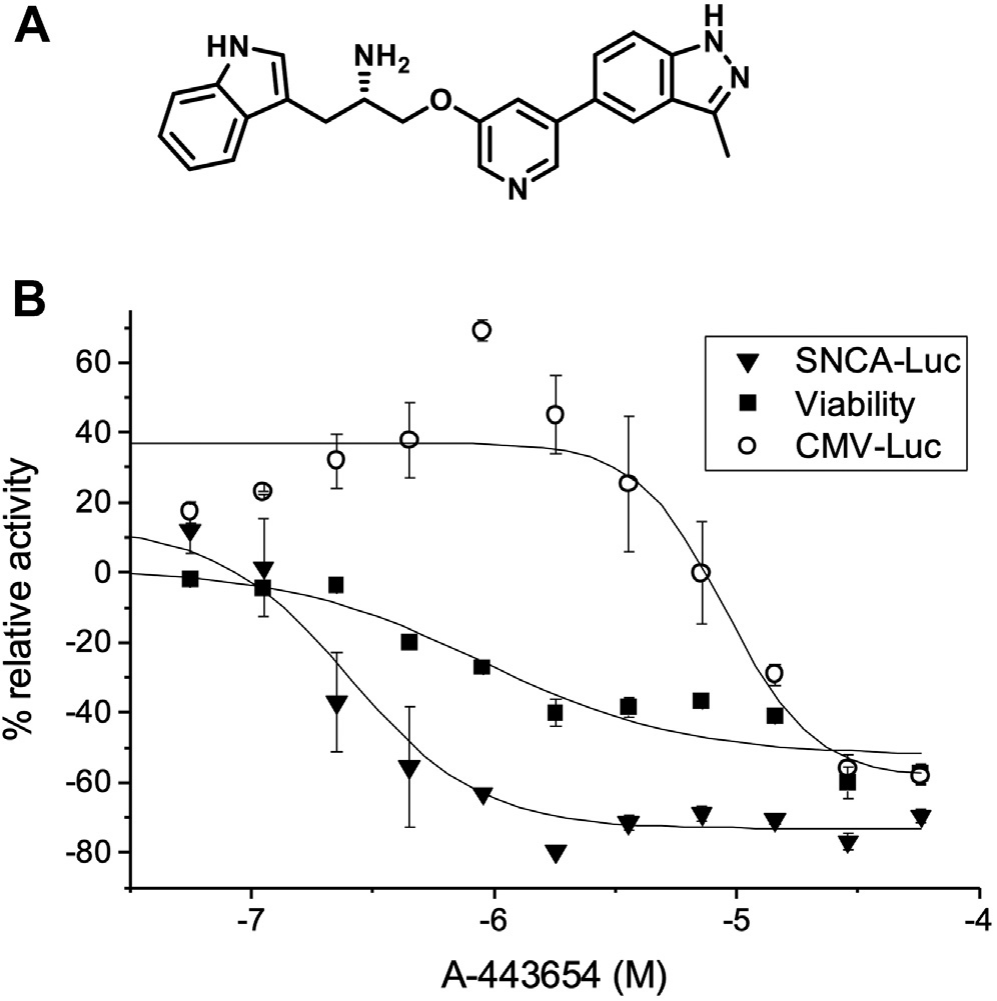
**Activity of A-443654 in quantitative high-throughput screen assays.***A*, molecular structure of A-443654. *B*, relative luciferase activity in SNCA-Luc (IC_50_ = 251 nM, *triangles*), CMV-luc (*open circles*), and viability (*squares*) of SNCA-luc cells measured after 24 h of treatment with A-443654 at the indicated doses.

We screened a total of 155,885 compounds, with 3 to 11 doses per compound depending on how the libraries were originally formatted, and measured the effects on SNCA-luc expression (21). The primary screen also included a viability measurement, using Gly-Phe-7-amino-4-trifluoromethylcoumarin (GF-AFC), and compounds were eliminated from further consideration if they were cytotoxic. The total number of active compounds passing cytotoxicity (cytotox) triage was 1996 (1.3%). The number of compounds screened, doses, and Z′ factors are provided in Table 1. After *in silico* analysis (clustering, removal of frequent hitter nuisance chemotypes), 842 compounds were selected for follow-up and retested at 11 doses in the SNCA-luc cells (luciferase and GF-AFC confirmation) and a CMV-luc expressing cell line to flag general transcriptional repressors. Following this triage, 33 compounds were tested by enzyme-linked immunosorbent assay (ELISA) to measure endogenous α-synuclein protein in 3X*SNCA* PD patient fibroblasts. Triaging of compounds through validation counter-screens and orthogonal assays is shown in Table 2. Five compounds were confirmed to reduce α-synuclein levels in secondary assays and by ELISA using patient cells, including NCGC00470280, NCGC00419055, NCGC00448911, NCGC00345809, and NCGC00347278 (A-443654) (Table 3). We eliminated NCGC00345809 from further consideration for having the least favorable cytotox and ELISA parameters (Table 3). Among the remaining compounds, A-443654 displayed the most promising profile when primary assays, ELISA, and Western blot results were considered collectively and was selected for further study. The structure of A-443654 is shown in Figure 1*A*. General transcriptional effects were not observed in the CMV-luc counter-screen (IC_50_ 8.91 μM) (Fig. 1*B*).

**Table 1:** Libraries, compound numbers, doses, Z′ factors, activities

Library	Compounds	Doses	Z' (SNCA-luc)	Active (SNCA-luc)	Passing cytotox triage
NPC^a^	2816	3	0.42	12	10
Epigenetics Focused Library	339	4	0.51	6	4
NPACT	5157	7	0.59	29	9
Genesis	96,260	4	0.6	1937	1562
PTL	3000	4	0.62	14	11
MIPE 4.0	1978	11	0.65	155	47
LOPAC	1280	4	0.60	18	15
Sytravon	45,055	4	0.63	2719	333
Sum for all libraries	155,885	--	--	4890	1996

The total number of compounds was 155,885.

a The NPC library included only three doses, so curve classes −1.1, −1.2, −2.1, −2.2 could not be computed. There were 12 compounds that showed inhibition (all negative curve classes).

**Table 2:** Triage of 842 compounds through validation counter-screens (SNCA-luc, CMV-luc, Cytotox) and orthogonal assays (ELISA)

Triage step	Criterion^a^	No. of Compounds
Total evaluated in follow-ups	1	842
Confirmed in the SNCA-luc assay (of 842)	2	446
Passing CMV-luc and Cytotox counter-screens (of 446)	3	33
Lowering SNCA in 3X*SNCA* fibroblasts by ELISA (of 33)	4	5

a Criteria for advancing compounds: (1) Negative curve class in primary screen. (2) Confirmed negative curve class, >30% inhibition for SNCA-luc assay. (3) ΔAUC (single metric of potency and efficacy) of >40 comparing SNCA-luc *versus* both CMV-luc and cytotox assays. (4) >50% reduction for SNCA at 1 μM.

**Table 3:** Lead compounds

Compound	MW	Function	Primary assay IC_50_ (μM)	SNCA efficacy (%)	Curve class	Cytotox IC_50_ (μM)^a^	Cytotox efficacy (%)^a^	Cytotox curve class	ELISA IC_50_^b^	α-Synuclein reduction at 0.5 μM (WB) (%)^c^
NCGC00470280	400.6	Unknown	15.8	−60	−2.4	n/a	n/a	4	30 nM	82
NCGC00419055	276.3	Unknown	15.8	−85	−1.2	n/a	n/a	4	366 nM	29
NCGC00345809	473.3	Cdk/Crk Inhibitor	0.11	−74	−1.2	0.09	−42	−1.2	411 nM	n.d.^d^
NCGC00448911	421.4	Unknown	15.8	−66	−1.2	n/a	n/a	4	99 nM	67
NCGC00347278 (A-443654)	397.5	AKT inhibitor	0.25	−86	−1.1	0.89	−53	−1.2	89 nM	85

a Designated n/a for inactive cytotox curve class of 4.

b *SNCA* triplicated human fibroblast cell line ND27760.

c HEK-293 cells.

d Value not determined.

### A-443654 effects on the AKT pathway

**Figure 2:**
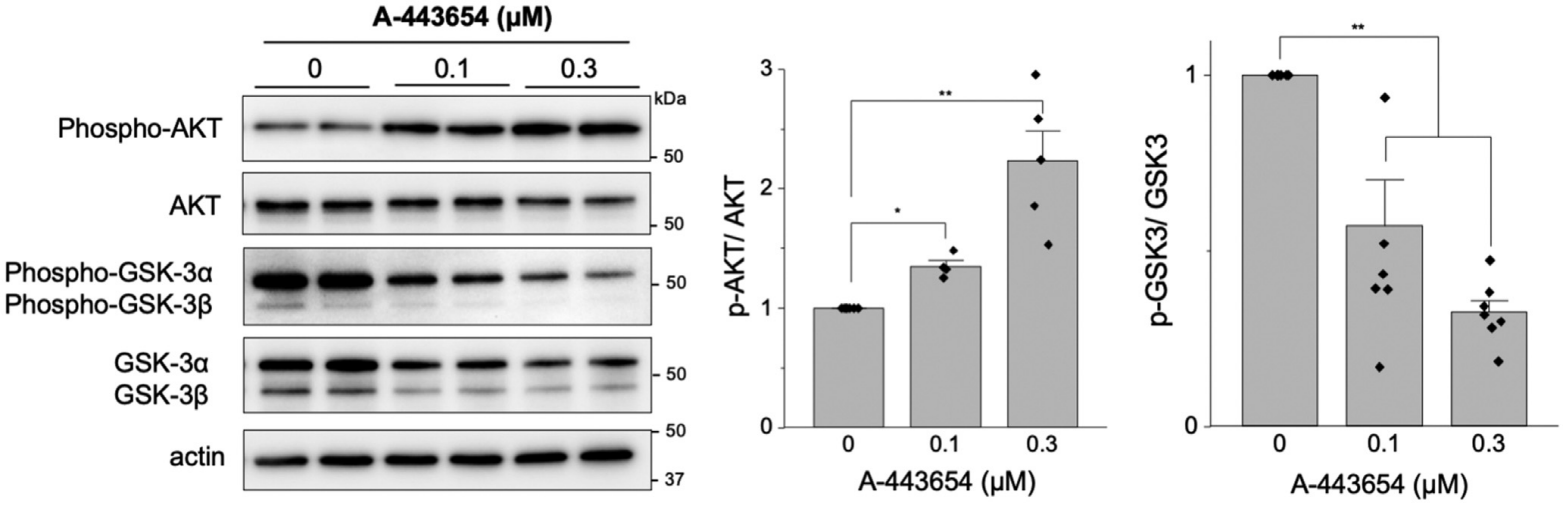
**A-443654 effects on the AKT pathway.** Western blot of phospho- and total AKT and phospho-GSKα/β in HEK-293 cells treated with increasing doses of A-443654 (*left*). Quantification of three independent experiments (*right*). ∗*p* < 0.05 and ∗∗*p* < 0.01, Student’s *t* test with Bonferroni correction.

A-443654 is a well-characterized inhibitor of the serine/threonine kinase Akt. Inhibition of Akt by A-443654 is associated with “paradoxical” Akt phosphorylation at Ser473, while effectively inhibiting its downstream effectors GSK3α/β, molecular target of rapamycin (mTOR), and TSC2 (22, 23, 24). In agreement with this, A-443654 increased AKT Ser473 phosphorylation in a dose-dependent manner in HEK-293 cells, which was associated with reduced total AKT protein abundance after extended exposure to the inhibitor (Fig. 2). Decreased phospho-GSK3α/β was observed in response to A-443654, consistent with inhibition of AKT signaling (Fig. 2).

### A-443654 reduces α-synuclein protein monomers, trimers, and mRNA levels

**Figure 3:**
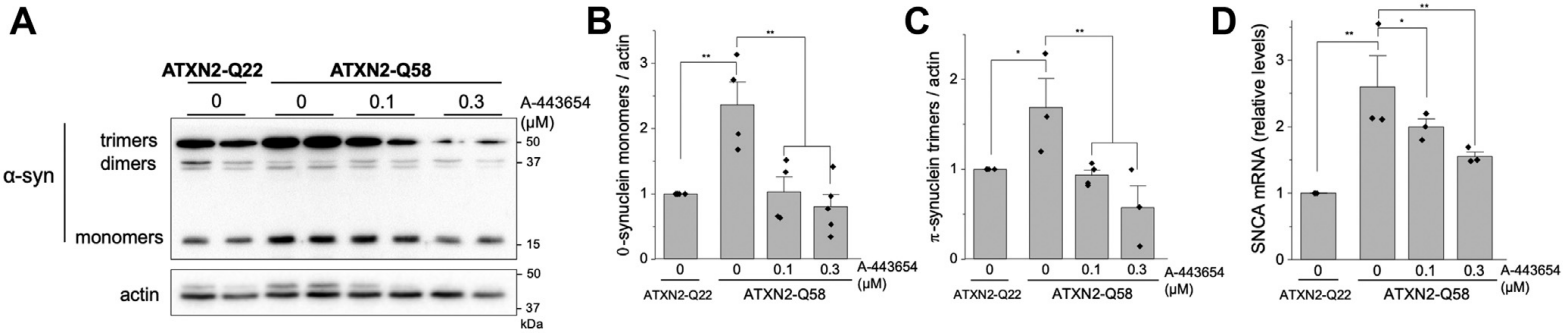
**α-Synuclein transcript and protein decrease in HEK-293 cells.***A*, western blot of HEK-293 cells expressing normal ATXN2 (ATXN2-Q22) and a glutamine-expanded pathologic variant (ATXN2-Q58) showing α-synuclein trimers, dimers, and monomers after 48 h of A-443654 treatment. Quantification of α-synuclein monomers (*B*) and trimers (*C*) from three independent experiments (*right*). *D*, *SNCA* mRNA levels quantified by qPCR. ∗*p* < 0.05 and ∗∗*p* < 0.01, Student’s *t* test with Bonferroni correction.

To characterize the effects of A-443654 on α-synuclein expression we used HEK-293 cells (ATXN2-Q22) in which the *ATXN2* allele was replaced with a pathogenic variant containing a CAG expansion of 58 repeats (ATXN2-Q58 cells). This cell line represents a model of spinocerebellar ataxia type 2 (25) useful for our testing because α-synuclein monomers, dimers, and trimers can readily be detected by Western blot (at estimated 17, 34 and 51 kDa, respectively) and its levels are highly increased. It had previously been established that the antibody we used is competent for detecting high-molecular-weight forms of α-synuclein (26, 27, 28, 29). To assure this, we knocked down *SNCA* expression by RNAi and observed no monomeric α-synuclein and the greatest reduction of dimeric and trimeric forms at the longest treatment time (Fig. S2). We observed elevated levels of α-synuclein protein monomers and trimers (2.5 ± 0.4-fold increase in monomers and 1.7 ± 0.3-fold increase in trimers) in ATXN2-Q58 cells compared with the isogenic parent cell line with a normal *ATXN2* (Fig. 3*A*). In addition, ATXN2-Q58 cells exhibited transcriptional upregulation of *SNCA* mRNA (Fig. 3*B*). We did not quantify α-synuclein dimers because they were of low abundance and exhibited high interexperiment variability. Treatment of ATXN2-Q58 cells with A-443654 for 48 h completely restored the levels of trimer and monomer α-synuclein proteins (Fig. 3*A* and *C*) and significantly reduced *SNCA* mRNA levels (Fig. 3*D*).

**Figure 4:**
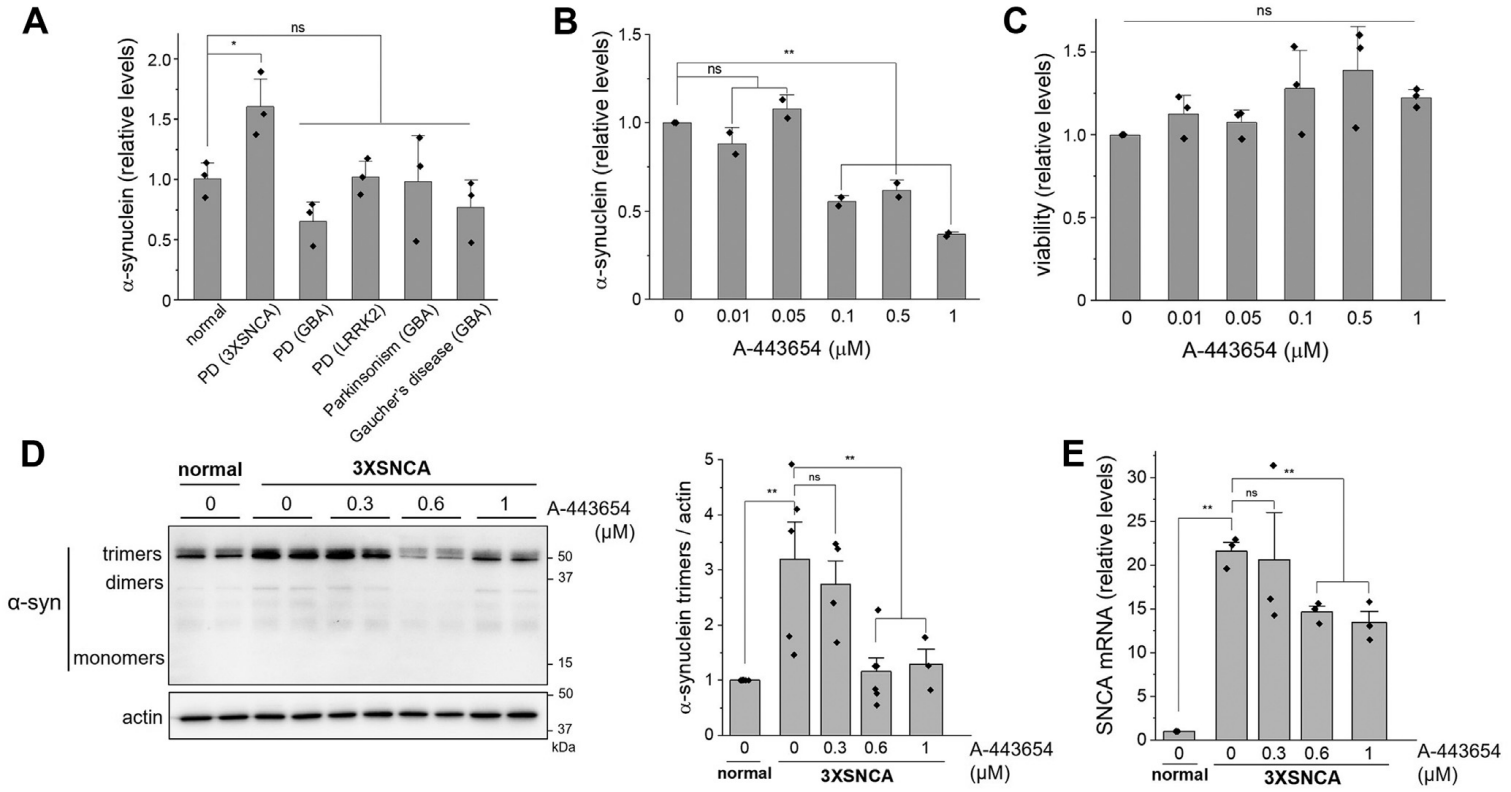
**Effects of A-443654 in 3X*SNCA* fibroblasts.***A*, relative levels of α-synuclein protein in fibroblasts from patients with no pathology (normal), PD (3X*SNCA*, *GBA* mutation, *LRKK2* mutation), parkinsonism (*GBA* mutation), or Gaucher’s disease (*GBA* mutation) quantified by ELISA. *B*, ELISA of α-synuclein levels in 3X*SNCA* fibroblasts treated with increasing doses of A-443654 for 48 h. *C*, viability of 3X*SNCA* fibroblasts treated with increasing doses of A-443654 for 48 h by MTT. *D*, western blot analysis of α-synuclein in normal and 3X*SNCA* fibroblasts (*left*) and quantification of trimers from three independent experiments (graph, *right*). *E*, qPCR quantification of *SNCA* mRNA levels in normal and 3X*SNCA* fibroblasts. ∗*p* < 0.05; ∗∗*p* < 0.01; ns, not significant. Student’s *t* test with Bonferroni correction.

We next studied skin fibroblasts from a patient with PD with a triplication of the *SNCA* gene (3X*SNCA* fibroblasts). Compared with skin fibroblasts from a healthy individual (normal) or patients with parkinsonism, Gaucher’s disease, or PD linked to mutations in the *GBA* or *LRKK2* gene, the 3X*SNCA* fibroblasts displayed between 1.7- and 2.1-fold increase in α-synuclein abundance (Fig. 4*A*). 3X*SNCA* fibroblasts treated with A-443654 for 48 h showed greater reduction of α-synuclein abundance at higher doses, as determined by ELISA (Fig. 4*B*), without cytotoxic effects (Fig. 4*C*). Western blotting analysis of 3X*SNCA* fibroblasts showed no detectable monomeric or dimeric forms of α-synuclein; however, α-synuclein trimers were elevated 3-fold and A-443654 successfully lowered them to levels comparable with normal fibroblasts (Fig. 4*D*). *SNCA* mRNA was highly increased in 3X*SNCA* fibroblasts (20-fold compared with normal control), and 1 μM A-443654 significantly reduced *SNCA* mRNA abundance by 38% (Fig. 4*E*).

### Reduction of α-synuclein in PD neurons

**Figure 5:**
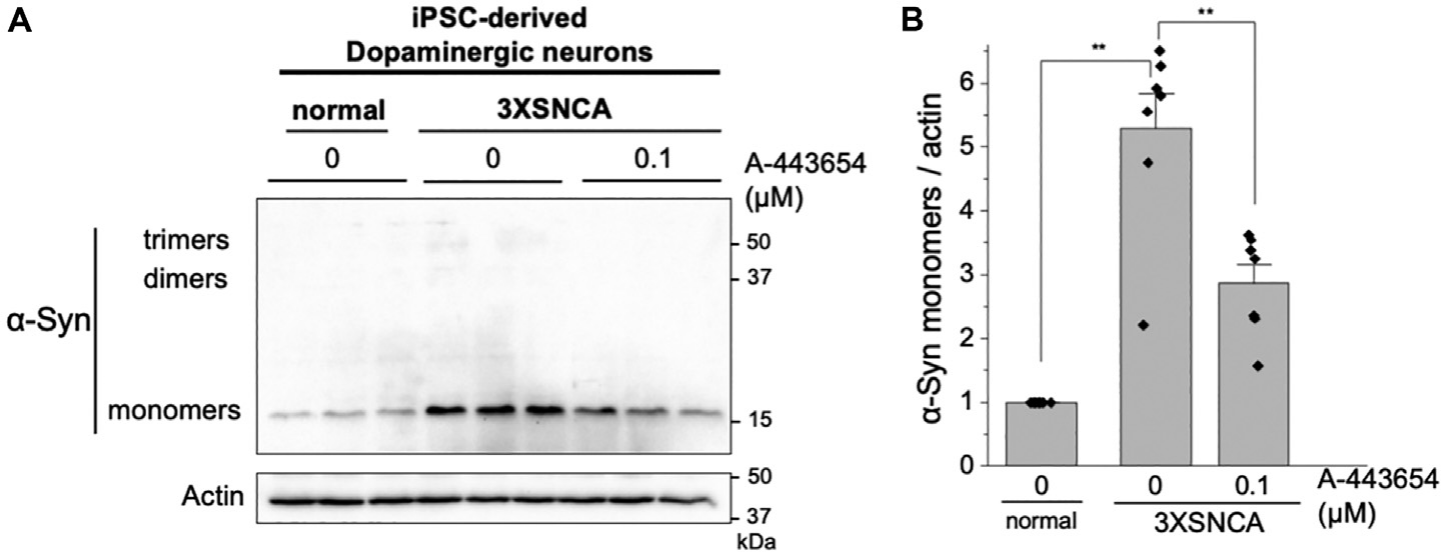
**A-443654 reduces α-synuclein in iPSC-derived dopaminergic neurons.***A*, western blot of normal and 3X*SNCA* iPSC-derived dopaminergic neurons showing the presence of α-synuclein monomers and the response to A-443654. *B*, quantification of the relative levels of α-synuclein monomers. ∗∗*p* < 0.01, Student’s *t* test with Bonferroni correction. iPSC, induced pluripotent stem cell.

We next examined the activity of A-443654 in dopaminergic neurons, the cell type most impacted in PD. iPSCs with a triplication of the *SNCA* gene were differentiated into dopaminergic neurons, which were positive for tyrosine hydroxylase and had neurite outgrowths characteristic of neurons (Fig. S3). The PD 3X*SNCA* neurons had a >5-fold increase in α-synuclein monomer expression compared with normal neurons, and 0.1 μM A-443654 treatment reduced the abundance of monomers by >3-fold after a 48-h treatment (Fig. 5). α-Synuclein dimers and trimers were not detected in these cells (Fig. 5).

### A-443654 normalized abnormal autophagy and ER stress markers

**Figure 6:**
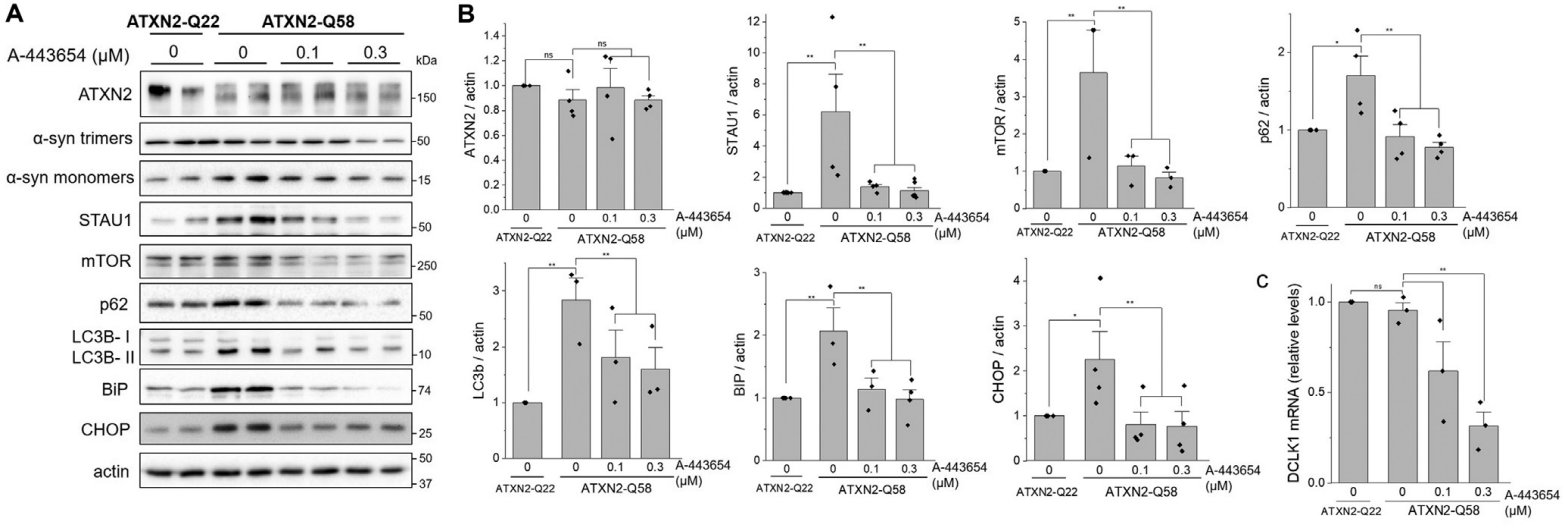
**A-443654 normalizes stress pathways in HEK-293 cells.** Western blots of ATXN2-Q58 cells for key cellular stress, autophagy, and UPR pathway markers (*A*) and quantification of three independent experiments (*B*). *C*, mRNA levels of *DCLK1* in ATXN2-Q22 and ATXN2-Q58 cells treated with A-443654 for 48 h ∗*p* < 0.05 and ∗∗*p* < 0.01, Student’s *t* test with Bonferroni correction.

Accumulation of α-synuclein leads to cellular stress and subsequent activation of signaling that can either promote survival or activate cell death pathways in the presence of overwhelming damage. We have previously described that ATXN2-Q58 cells display significant increases in unfolded protein response (UPR) mediators and autophagic markers, as well as the RNA-binding protein STAU1, which can enhance the detrimental effects of ATXN2 expansions (30). Upon treatment with A-443654, decreases in α-synuclein levels were associated with normalized STAU1 abundance (Fig. 6*A*). In addition, autophagy mediators mTOR, p62, and LC3-II and UPR factors BiP and CHOP returned to baseline levels, indicating A-443654 efficiently restored protein homeostasis (Fig. 6*A*). The expression of ATXN2 was unchanged, indicating that the effects of A-443654 are independent of ATXN2 (Fig. 6*A*).

Previous studies revealed that knockdown of doublecortin like kinase 1 (*DCLK1*) reduced α-synuclein level and toxicity (31). Therefore, we investigated whether A-443654 could modulate DCLK1 in our ATXN2-Q58 model. We found that A-443654 dose dependently decreased the levels of *DCLK1* mRNA (Fig. 6*B*), suggesting this pathway could be recruited by A-443654 to reduce α-synuclein.

## Discussion

We performed a high-throughput screen of 155,885 compounds at multiple doses using a genome-edited cell line that expresses α-synuclein fused to luciferase under native expression control. We identified A-443654, a pan AKT inhibitor as a potent inhibitor of α-synuclein transcription that normalized the abundance of monomeric and/or oligomeric forms of α-synuclein in ATXN2-Q58 HEK-293 cells, 3X*SNCA* fibroblasts, and 3X*SNCA* iPSC-derived dopaminergic neurons. In addition, A-443654 normalized autophagy dysfunction and ER stress in ATXN2-Q58 cells. A-443654 may represent a useful scaffold for developing an effective therapeutic for PD and may also serve as a tool for further interrogating the contributions of AKT signaling on PD pathogenesis.

Other efforts to silence *SCNA* expression include oligonucleotide-based therapeutics (antisense oligonucleotides and RNAi) and the development of small molecules lowering *SNCA* transcription, translation, or posttranslational half-life. Reduction of *SNCA* expression in PD mouse models was achieved using antisense oligonucleotide, which improve motor control and other phenotypes (32, 33). Reduction of *SNCA* expression has also been demonstrated in PD mice using siRNAs or shRNA plasmids, and efficacy was enhanced when complexing therapeutic nucleic acids with nanoparticles (34, 35, 36). An adeno-associated virus–delivered *SNCA* shRNA improved behavioral phenotypes in a rat PD model, but these effects were confounded by neuronal toxicity (37).

Several small molecules have also been implicated in modulating SNCA expression. A small molecule screen of 1124 compounds identified multiple β2-adrenoreceptor agonists that lower *SNCA* mRNA abundance (38), resulting in an epidemiologic study of nearly 2 million adults that indicated that the use of β2-adrenoreceptor agonists could significantly reduce the risk of PD (39). Posiphen is another compound that has been proposed for PD, as it inhibits 5′-UTR-mediated *SNCA* translation (40) and can alter the expression of *SNCA* in the gut of mice (41). Posiphen has been evaluated in phase I clinical trial for Alzheimer’s disease as it was also shown to modify accumulation of amyloid-β (42). The drug Nilotinib, which acts by inducing autophagic degradation of α-synuclein, is the only small molecule that has been evaluated in clinical trials for PD (43, 44). *In vivo* use of A-443654 was previously reported in mice, and when delivered by subcutaneous injection, it reached plasma and tissue concentrations above 0.5 μM and effectively inhibited mTOR phosphorylation in human prostate tumor xenografts (22). Of interest, A-443654 can also activate mTORC2, which in turn phosphorylates the functionally inactive A-443654-bound AKT on Ser 473 in a compensatory manner (23). Thus, A-443654 is considered not only an AKT inhibitor but also an mTORC2 activator. In an Angelman syndrome mouse model with overactive mTORC1 and underactive mTORC2, treatment with A-443654 restored the mTORC1/mTORC2 balance and rescued abnormal long-term potentiation in CA1 hippocampal neurons (45). This is highly relevant because mTORC1/mTORC2 imbalance was seen previously in PD and mTOR is considered a therapeutic target for PD (46, 47). Although A-443654 is often described as a selective AKT inhibitor and its effect on the AKT/mTOR pathway is well established, A-443654 may also block the ATP-binding pocket of other kinases and thus we cannot rule out that its effects on α-synuclein are exclusively AKT dependent (48).

Because of its actions at both the mRNA and protein levels, A-443654 may be particularly useful for normalizing neuronal damage in PD and related α-synucleinopathies. We found that the reduction of α-synuclein with A-443654 simultaneously normalized autophagy and ER stress pathways. The autophagy outcome likely relates to A-443543 normalizing mTOR downstream of AKT, which is a potent autophagy inhibitor. A-443654 also lowered STAU1 abundance, which directly binds the *MTOR* transcript to increase its translation (49, 50). We then investigated DCLK1 because a recent study showed that targeting this kinase could normalize α-synuclein levels by inducing autophagy, preserving the viability of dopaminergic neurons in the substantia nigra pars compacta in two different mouse models of PD (31). We found that A-443654 reduced *DCLK1* transcript levels, constituting the first report of a small molecule capable of modifying the expression of this gene. We conclude that A-443654 lowers *SNCA* expression and normalizes autophagy and ER stress, which additionally facilitates clearance of pathogenic α-synuclein protein.

The pharmacology of A-443654 on PD pathways highlights its potential as a preventative of the multifactorial cascades that are responsible for the death of dopaminergic neurons. A-443654 effects on *SNCA*, AKT, *DCLK1*, autophagy, and the UPR suggest it may have broad beneficial actions. This study adds to a body of research focusing on modulating α-synuclein expression to treat PD, presenting A-443654 as an additional lead toward restoring cellular functions impaired in PD and related α-synucleinopathies. Full preclinical characterization, including *in vitro* and *in vivo* toxicological studies would be needed to ensure the suitability of A-443654 for clinical trials.

## Experimental procedures

### Cell culture for primary and counter-screening assays

The primary screening assay cell line (HEK-293-SNCA-luc) was generated as described (19). Briefly, we modified the *SNCA* gene in HEK-293 cells using genome editing (zinc-finger nuclease) to insert the luciferase gene (*luc*+) downstream and in frame with the *SNCA* coding sequence. The counter-screening assay cell line was generated by transfecting HEK-293 cells with pGL2h-CMV-luc (HEK-293-CMV-luc) and selecting with hygromycin. Cultures were maintained as mixed populations of transfected cells (clonal colony selection was not done). Both cell lines were maintained in Dulbecco's modified Eagle medium (DMEM) with 10% fetal bovine serum and 1× penicillin/streptomycin. HEK-293-SNCA-luc cells were cultured in media containing 10 μg/ml puromycin, and HEK-293-CMV-luc cells were cultured in media containing 40 μg/ml hygromycin.

### CRISPR interference

Plasmid pHR_SFFV-dCas9_BFP_KRAB (Addgene #46911) encoding a nuclease dCas9 tethered to the KRAB transcriptional repressor was cotransfected into HEK-293-SNCA-luc cells along with a plasmid expressing a single guide RNA (sgRNA) directing dCas9-KRAB localization just downstream of the *SNCA* transcriptional start site. The sequence 5′-CGCACCTCACTTCCGCGTCG-3′ (corresponding to nucleotides 88–107 in exon 1 of the *SNCA* gene) was cloned into pGS-gRNA plasmid (Genscript). Transfections were performed in 96-well format. Plasmid DNA (35 ng of each plasmid, 70 ng total, in 10 μl OptiMEM) and Lipofectamine 2000 (ThermoFisher, 0.25 μl lipid in 10 μl OptiMEM) were incubated at room temperature for 20 min, combined, and added to the plate (20 μl volume). Next, 50 μl of cells suspended in DMEM (without antibiotics) at 1 × 10^6^ cells/ml was added to each well (50,000 cells). After overnight incubation, an additional 50 μl of complete medium (DMEM with 10% fetal bovine serum, 1× penicillin/streptomycin) was added to each well. Plates were sealed with a Breathe-easy film (Diversified Biotech) and returned to a 37 °C incubator (5% CO_2_, 95% RH). Following 120 h, 100 μl of ONE-Glo reagent (Promega) was added to each well, the plate was incubated for 10 min at room temperature, and luminescence was measured using a ViewLux (PerkinElmer) equipped with clear filters.

### Libraries

Compound libraries used included the following: NCATS Pharmaceutical Collection (NPC, 2400 compounds, approved by US Food and Drug Administration or related agencies in other countries), Genesis Chemical Library (96,260 compounds, library that emphasizes high-quality chemical starting points, sp3-enriched chemotypes, and core scaffolds that enable rapid derivatization *via* medicinal chemistry), NCATS Pharmacologically Active Chemical Toolbox (NPACT, 5157 compounds, annotated compounds that inform on biological pathways and cellular processes), PTL (3000 compounds), Mechanism Interrogation PlatE (MIPE 4.0, 1978 compounds, oncology-focused collection), LOPAC^1280^ collection of pharmacologically active compounds (Sigma, 1280 compounds), Sytravon (45,056 compounds, retired Pharma screening collection that contains a diversity of novel small molecules, with an emphasis on medicinal chemistry-tractable scaffolds). The total number of compounds was 155,885. All compound solutions were prepared in dimethyl sulfoxide (DMSO) solvent.

### Primary quantitative high-throughput screen

A total of 1500 HEK-293-SNCA-luc cells were seeded into each well of a 1536-well white tissue culture treated plate, at 4 μl per well using phenol red–free DMEM (4.5 g/l glucose, 25 mM Hepes, cat #21063 [Thermo]). After 24 h at 37 °C, 23 nl compounds were transferred *via* a Kalypsys pin tool equipped with a 1536-slotted pin array. After incubation at 37 °C for 24 h, 1 μl of Gly-Phe-7-amino-4-trifluoromethylcoumarin (GF-AFC, prepared at 125 μM in PBS) was added, plates were incubated for 30 min, and fluorescence was measured using a ViewLux microplate reader (PerkinElmer) equipped with ex: 405/10 and em: 540/25 filters. Next, ONE-Glo or Steadylite plus (PerkinElmer) luciferase detection reagent (3 μl) was added to each well and incubated for an additional 15 min. Luminescence is then measured on the ViewLux imager equipped with clear filters. All screening operations were performed on a fully integrated robotic system (Kalypsys). Vehicle-only plates, with DMSO being pin transferred to every well, were inserted at the beginning of screening runs to confirm expected assay performance. For both the luciferase and GF-AFC assays, activity was normalized to wells containing medium only (−100% activity, full inhibition) and SNCA-luc cells treated with DMSO vehicle control (0% activity), contained on the same plate as test samples. Dose–response curves were fit using the Hill equation, and then curve classes were determined as described, where 1 = complete response, 2 = incomplete response, 3 = single point activity, and 4 = inactive (21). Compounds in curve classes −1.1, −1.2, −2.1, −2.2 in the SNCA-luc assay were considered active. Compounds were eliminated from further consideration if also active (curve class −1.1, −1.2, −1.3, −1.4, −2.1, −2.2, −2.3, −2.4) in the GF-AFC cytotoxicity assay.

### Luciferase counter-screen assays

Compounds exhibiting inhibitory activity (defined as curve class −1.1, −1.2, −2.1, −2.2) were identified, then manually curated by structure and activity. A total of 842 compounds were selected for follow-up and evaluated in the same manner as in the primary assay but using HEK-293 cells stably expressing CMV-luc. Compounds were considered as active if they inhibit SNCA-luc in the primary assay but did not inhibit luciferase in CMV-luc counter-screen.

### Generation of HEK-293 with the ATXN2-Q58 allele

HEK-293 cells with an expansion in the *ATXN2* gene CAG repeat to 58 CAGs, designated ATXN2-Q58 cells, were generated by CRISPR/Cas9 genome editing, as described (25). The parental HEK-293 cell line was designated as ATXN2-Q22 to note *ATXN2* was not modified in this line. Cells were maintained in DMEM (ThermoFisher #11965118) with 10% FBE (FB essence, Avantor Seradigm) and 1× penicillin/streptomycin (ThermoFisher).

### Skin cell fibroblast cell lines and culture conditions

Skin cell fibroblasts used in the study were ND27760 (*SNCA* triplication, Park1, 55-year-old female patient diagnosed with PD), ND31630 (GBA[N370S], 69-year-old male patient diagnosed with parkinsonism), ND34235 (LRRK2[G1019S], Park8, 71-year-old male patient diagnosed with PD), GM10915 (GBA, 7-year-old male patient diagnosed with Gaucher’s disease type 1), ND29756 (GBA[N370S], 55-year-old female patient diagnosed with PD), and ND34770 (normal, 72-years-old woman with no diagnosis). Cell lines were obtained from the Coriell Institute or the NINDS Human Cell and Data Repository and were cultured in DMEM (ThermoFisher #11965118) with 15% fetal bovine essence (FBE), 15 mM Hepes, 1× penicillin/streptomycin, and 1× nonessential amino acids. Experiments were carried out in 10% FBE.

### Production of dopaminergic neurons

The iPSC line ND34391 was obtained from the NINDS Human Cell and Data Repository. This line was derived from the same individual as the patient-derived skin cell fibroblast line ND27760, which has a triplication of the *SNCA* gene. Control iPSCs were produced from BJ fibroblasts by transfecting an episomal plasmid expressing the four reprogramming factors OCT4, c-MYC, SOX2, and KLF4. iPSCs were cultured in E8 flex media (ThermoFisher A2858501) over a substrate of vitronectin at 1 μg/cm^2^. Differentiation of iPSCs to dopaminergic neurons was accomplished using the PSC Dopaminergic Neuron Differentiation Kit (ThermoFisher A3147701) according to the manufacturer’s instructions. Differentiation steps were verified with the Human Dopaminergic Neuron Immunocytochemistry Kit (A29515, ThermoFisher) and by Western blotting to verify expression of tyrosine hydroxylase. For treatment, neurons were seeded at 2 × 10^5^ cells/cm^2^, and after 2 weeks of maturation A-443654 was added to the cell culture medium at the indicated doses. Neurons were collected after 48 h, and lysates were prepared in RIPA buffer (Pierce, ThermoFisher 89900) with protease inhibitors (Complete Protease Inhibitor, Roche).

### ELISA assays

ELISA assays were performed using the LEGEND MAX Human α-Synuclein ELISA Kit following the vendor’s instructions (BioLegend), with triplicate assays. Values were calculated as means and standard deviations (SDs) of pg/ml per mg of protein or of percent of control. Chemiluminescence was read in 96-well plates using a Beckman 880 DTX multimode plate reader.

### Western blotting

Proteins were separated on precast polyacrylamide gels (Bio-Rad) and transferred to Hybond (Amersham). For detection of α-synuclein, proteins were cross-linked to membranes using 0.4% paraformaldehyde (51, 52). Chemiluminescent detection was performed with enhanced chemiluminescence (Amersham). Antibodies used were anti-α-synuclein antibody ([1:1500], BD Transduction Laboratories, 610787), anti-Ataxin-2 antibody ([1:4000], BD Biosciences, 611378), anti-Staufen antibody ([1:5000], Novus Biologicals, NBP1-33202), anti-BiP antibody ([1:3000], Genetex, GTX113340), anti-CHOP antibody ([1:3000], Cell Signaling Technology, 2895), anti-mTOR antibody ([1:4000], Cell Signaling Technology, 2972), anti-SQSTM1/p62 antibody ([1:4000], Cell Signaling Technology, 5114), anti-LC3B antibody ([1:7000], Novus Biologicals, NB100-2220SS), anti-tyrosine hydroxylase antibody ([1:2000], Millipore-Sigma, AB152), and peroxidase-conjugated monoclonal anti-β-Actin antibody (clone AC-15) ([1:20,000], Sigma-Aldrich, A3854). The secondary antibody was peroxidase-conjugated anti-rabbit (Jackson ImmunoResearch Laboratories).

### RNA interference

RNAi assays utilized *SNCA* siRNA (Ambion Life Technologies AM16708 Silencer siRNA ID 217011) and a control siRNA (Qiagen All Star Negative Control siRNA 1027280). SiRNAs were used at 50 nM and were transfected into HEK-293 cells using Lipofectamine 2000 (ThermoFisher). Cells were harvested 2 and 5 days post transfection.

### Viability assay

Viability assays performed during high-throughput screening were described above. In subsequent experiments viability was determined using the CellTiter 96 Non-Radioactive Cell Proliferation Assay (3-(4,5-dimethylthiazol-2-yl)-2,5-diphenyltetrazolium [MTT]) following the vendor’s instructions (Promega), in 96-well plates. Optical density was measured at a wavelength of 595 using a Beckman 880 DTX multimode plate reader.

### Quantitative real-time PCR

Total RNA was extracted from cells cultured in 6-well plates using the RNeasy Mini Kit (Qiagen) according to the manufacturer’s protocol. The extracted RNA was then used to synthesize cDNA using ProtoScript First Strand cDNA Synthesis Kit (New England Biolabs). Quantitative real-time PCR (qPCR) was performed in an Applied Biosystems QuantStudio 12K Flex Real-time PCR system using TaqMan Gene Expression Assays (FAM) (ThermoFisher, SNCA Hs00240906_m1, DCLK1 Hs00178027_m1, ACTB Hs01060665_g1) and TaqMan Fast Advanced Master Mix (ThermoFisher). PCR cycling parameters were set according to the manufacturer’s protocol for TaqMan Fast Advanced Master Mix and TaqMan Gene Expression Assays. The qPCR data were analyzed by the ΔΔCt method, using ΔΔCt values calculated by the QuantStudio 12K Flex software.

### Statistical analysis

To maintain statistical rigor, all Western blot and qPCR experiments included independent replication. Data were analyzed and plotted using Excel or GraphPad Prism. T tests were two tailed with equal variances assumed. Post hoc tests following analysis of variance (ANOVA) used the Bonferroni correction.

## Data availability

All screening data are available in PubChem (AID 1671193 and AID 1671194).

## Supporting information

This article contains supporting information.

## Conflict of interest

The authors declare that they have no conflicts of interest with the contents of this article.
