# Supplementary material for: The AKT modulator A-443654 reduces α-synuclein expression and normalizes ER stress and autophagy
Source: J Biol Chem. 2021 Sep 11;297(4):101191. doi: 10.1016/j.jbc.2021.101191 (PMC8482485; doi:10.1016/j.jbc.2021.101191)
Supplement: Supplemental Figures S1–S3 [file mmc1.pdf]

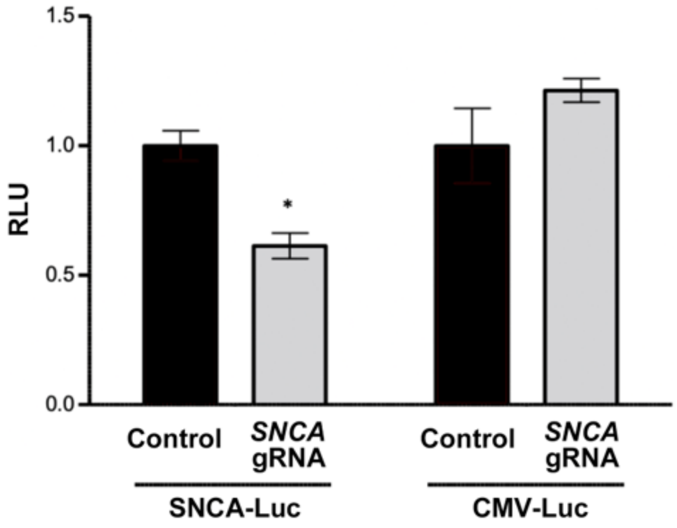

Supplemental Fig. 1. Validation of screening cell lines using CRISPRi. Normalized RLU of untransfected cells (control) or cells transfected with a guide RNA positioning the dCAS9-KRAB complex to just downstream of the *SNCA* transcriptional start site (*SNCA* gRNA). (*=p < 0.05, Student's t-test).

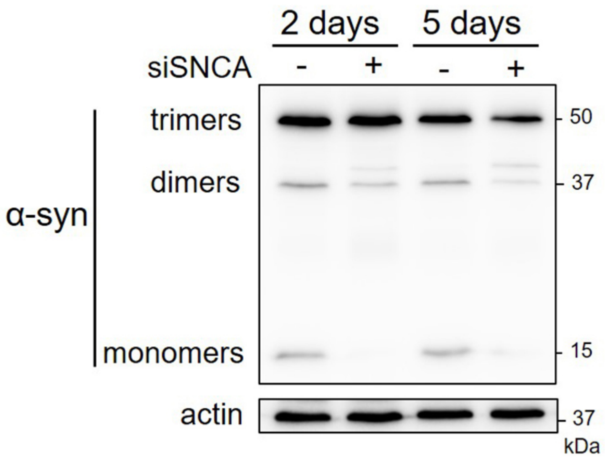

Supplemental Fig. 2. Validation of α-Synuclein antibody. HEK-293 cells were transfected with *STAU1* siRNA for 2 or 5 days and α-synuclein was detected using α-synuclein antibody. Turnover of aggregated α-synuclein (trimers) was observed for the 5 day timepoint.

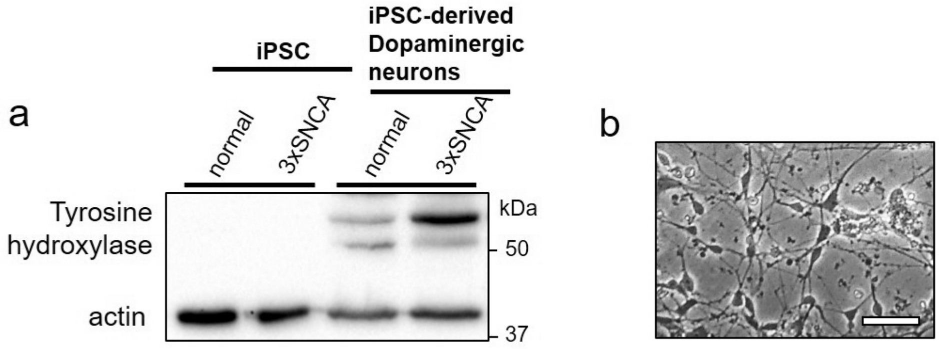

Supplemental Fig. 3. Generation of iPSC-derived dopaminergic neurons. (a) Western blot for tyrosine hydroxylase in extracts from iPSCs and iPSC-derived dopaminergic neurons. (b) Phase contrast image of iPSC-derived dopaminergic neurons. Scale bar, 50 μm.
